# Large-Scale Internetwork Functional Connectivity Mediates the Relationship between Serum Triglyceride and Working Memory in Young Adulthood

**DOI:** 10.1155/2020/8894868

**Published:** 2020-11-01

**Authors:** Chunli Wang, Huanhuan Cai, Xuetian Sun, Li Si, Min Zhang, Yuanhong Xu, Yinfeng Qian, Jiajia Zhu

**Affiliations:** ^1^Department of Clinical Laboratory, The First Affiliated Hospital of Anhui Medical University, Hefei 230022, China; ^2^Department of Radiology, The First Affiliated Hospital of Anhui Medical University, Hefei 230022, China

## Abstract

Previous research has demonstrated that serum lipid profile is associated with cognitive function as well as brain structure and function in middle-aged, elderly, and clinical populations. However, the nature and extent of lipids-brain-cognition relationships in young adulthood are largely unknown. In this study, 157 healthy young adults underwent resting-state functional MRI scans. Functional connectivity between and within 14 functional networks were calculated using independent component analysis. Peripheral venous blood samples were collected to measure serum lipids. Working memory was assessed using a 3-back task. Linear regression, correlation, and mediation analyses were conducted to test for potential associations between serum lipids, inter- and intranetwork functional connectivity, and working memory performance. We found that higher serum triglyceride (TG) level was correlated with stronger connectivity between left frontoparietal and ventral attention networks, between right frontoparietal and dorsal attention networks, between right frontoparietal and dorsal sensorimotor networks, between right frontoparietal and lateral visual networks, and between salience (SN) and ventral sensorimotor (vSMN) networks, as well as lower connectivity between posterior default mode and left frontoparietal networks, between left frontoparietal and medial visual networks, and between ventral attention and dorsal sensorimotor networks. In addition, higher SN-vSMN connectivity was related to lower 3-back accuracy. More importantly, the relationship between serum TG and 3-back accuracy was mediated by SN-vSMN connectivity. Our findings not only may expand existing knowledge regarding serum lipids-brain-cognition relations from the perspective of large-scale functional network organization but also may inform a translational conceptualization of how to improve cognitive function through regulating serum lipids.

## 1. Introduction

Evidence is now emerging that serum lipid profile is linked to many aspects of human cognition. A large number of cross-sectional studies have established that higher serum levels of total cholesterol (TC), triglyceride (TG), and low-density lipoprotein cholesterol (LDL-C) are associated with poorer cognitive performance in multiple domains (e.g., working memory, recognition memory, executive control, sustained attention, and inhibitory processing) [1–5], whereas higher serum high-density lipoprotein cholesterol (HDL-C) level is related to better cognitive functions such as verbal learning, memory, and executive function [5–7]. These associations are also confirmed by longitudinal studies consistently suggesting that elevated TC, TG, and LDL-C in midlife are predictive of greater cognitive decline in late life while higher HDL-C predicts better maintenance of cognitive function [8–10]. However, prior research has focused mainly on examining serum lipids-cognition relationships in middle-aged, elderly, or clinical populations and placed less emphasis on exploring the nature and extent of such relations in young adulthood.

The brain has a very high lipid content that provides structural integrity and modulates fluidity of neuronal cells. Cholesterol is thought to play a vital role in neuronal structure and function [11], as it is not only a major component of the cell membrane but also a biosynthetic precursor of many important bioactive molecules. There have been several neuroimaging studies attempting to clarify the associations between serum lipids and the brain in both normal and disease-affected populations. As a consequence, previous findings consistently suggest a negative effect of serum TC, TG, and LDL-C yet a protective effect of HDL-C on brain structure and function, by using structural magnetic resonance imaging (MRI) to evaluate gray matter morphology [5, 12–14] and using functional MRI (fMRI) to measure task-induced brain activation [15] and resting-state functional connectivity [4, 16, 17]. Nevertheless, whether and how differences in serum lipid parameters relate to variations in neuroimaging phenotypes in healthy young adults is still an open question.

It is generally accepted that the brain is a complex system consisting of multiple functional networks subserving different functions [18–21]. Each functional network is composed of several brain regions with similar patterns of signal change over the course of resting-state fMRI, whereas different networks show different activity patterns. These functional networks can be automatically identified by independent component analysis (ICA) of resting-state fMRI data, a useful method enabling data-driven, exploratory investigation of temporal correlations among brain regions at rest [22, 23]. Examinations of functional connectivity between and within functional networks have largely improved our understanding of the large-scale functional organization in normal and abnormal brains [24–27]. However, little is known, so far, about the relationships between serum lipid levels and inter- and intranetwork functional connectivity.

In the present study, the first objective was to assess the relationships between serum lipids and inter- and intranetwork functional connectivity in a sample of 157 healthy young adults with a combination of resting-state fMRI data and ICA approach. The second purpose was to test the links between serum lipid-sensitive functional connectivity markers and working memory performance measured by a 3-back task. Finally, we aimed to characterize the meditative role of serum lipid-linked functional connectivity in accounting for the effects of serum lipids on working memory.

## 2. Materials and Methods

### 2.1. Participants

A total of 157 healthy young adults were recruited by advertisement. All participants met the inclusion criteria of Chinese Han, right handedness, and within a restricted age range of 18-30 years that corresponds to a period after the completion of major neurodevelopment and before the onset of neurodegenerative changes. Exclusion criteria included neuropsychiatric or severe somatic disorder, a history of alcohol or drug abuse, regular smoker, current medication (e.g., antibiotics or sedative hypnotics) within a month, pregnancy, MRI contraindications, and a family history of psychiatric illness among first-degree relatives. The MINI-International Neuropsychiatric Interview (M.I.N.I.) and Alcohol Use Disorders Identification Test (AUDIT) were used in the process of excluding participants. This study was approved by the ethics committee of The First Affiliated Hospital of Anhui Medical University. Written informed consent was obtained from all participants after they had been given a complete description of the study. Demographic and behavioral data of the sample are listed in Table 1.

### 2.2. Blood Sampling and Serum Lipid Measurement

After an overnight fasting period, peripheral venous blood samples (2 ml) were collected from all the participants in the morning. Samples were centrifuged to separate the serum at 3000 rpm for 10 minutes at room temperature, and fresh serum was used immediately for the analysis of lipid profile. The estimation of serum TC, TG, and HDL-C levels was carried out in an automated clinical auto analyzer (Roche Cobas 8000). Serum LDL-C level was estimated with the Friedewald equation [28].

### 2.3. Working Memory Assessment

The letter 3-back task was conducted on a computer to assess working memory [29] using E-Prime 2.0 (http://www.pstnet.com/eprime.cfm). During the task, each participant viewed a series of letters that were presented sequentially, and the presentation time of each letter stimulus was 200 ms with an interstimulus interval of 1800 ms. Participants were instructed to press a button on the right with their middle fingers if the letter that appeared on the screen was identical to the one presented 3 letters earlier and otherwise to press a button on the left with their index fingers. The task consisted of 60 trials. Before the formal test, participants were verbally instructed and had a practice test to ensure that they understood the task. The accuracy and mean reaction time of correct responses were used as the indices of working memory performance.

### 2.4. MRI Data Acquisition

MRI scans were obtained using a 3.0-Tesla MR system (Discovery MR750w, General Electric, Milwaukee, WI, USA) with a 24-channel head coil. Earplugs were used to reduce scanner noise, and tight but comfortable foam padding was used to minimize head motion. High-resolution 3D T1-weighted structural images were acquired by employing a brain volume (BRAVO) sequence with the following parameters: repetition time (TR) = 8.5 ms; echo time (TE) = 3.2 ms; inversion time (TI) = 450 ms; flip angle (FA) = 12°; field of view (FOV) = 256 mm × 256 mm; matrix size = 256 × 256; slice thickness = 1 mm, no gap; 188 sagittal slices; and acquisition time = 296 s. Resting-state blood-oxygen-level-dependent (BOLD) fMRI data were acquired using a gradient-echo single-shot echo planar imaging (GRE-SS-EPI) sequence with the following parameters: TR = 2000 ms, TE = 30 ms, FA = 90°, FOV = 220 mm × 220 mm, matrix size = 64 × 64, slice thickness = 3 mm, slice gap = 1 mm, 35 interleaved axial slices, 185 volumes, and acquisition time = 370 s. Before the scanning, all subjects were instructed to keep their eyes closed, relax, move as little as possible, think of nothing in particular, and not fall asleep during the scans. During and after the scanning, we asked subjects whether they had fallen asleep to confirm that none of them had done so. All MR images were visually inspected to ensure that only images without visible artifacts, lesions, and regional deformations were included in subsequent analyses.

### 2.5. fMRI Data Preprocessing

Resting-state BOLD data were preprocessed using Statistical Parametric Mapping software (SPM12, http://www.fil.ion.ucl.ac.uk/spm) and Data Processing & Analysis for Brain Imaging (DPABI, http://rfmri.org/dpabi) [30]. The first 10 volumes for each participant were discarded to allow the signal to reach equilibrium and the participants to adapt to the scanning noise. The remaining volumes were corrected for the acquisition time delay between slices. Then, realignment was performed to correct the motion between time points. Head motion parameters were computed by estimating the translation in each direction and the angular rotation on each axis for each volume. All participants' BOLD data were within the defined motion thresholds (i.e., translational or rotational motion parameters less than 2 mm or 2°). We also calculated frame-wise displacement (FD), which indexes the volume-to-volume changes in head position. In the normalization step, individual structural images were firstly coregistered with the mean functional image; then, the transformed structural images were segmented and normalized to the Montreal Neurological Institute (MNI) space using a high-level nonlinear warping algorithm, that is, the diffeomorphic anatomical registration through the exponentiated Lie algebra (DARTEL) technique [31]. Finally, each filtered functional volume was spatially normalized to MNI space using the deformation parameters estimated during the above step and resampled into a 3-mm cubic voxel. After spatial normalization, all data sets were smoothed with a Gaussian kernel of 6 × 6 × 6 mm^3^ full-width at half maximum (FWHM).

### 2.6. Independent Component Analysis

ICA was conducted to parcellate the preprocessed fMRI data with the GIFT toolbox (http://mialab.mrn.org/software/gift/), and the number of independent components (*N* = 26) was estimated automatically by the software. Spatial ICA decomposes the participant data into linear mixtures of spatially independent components that exhibit a unique time course profile. This was achieved by using two data reduction steps. First, principal component analysis was applied to reduce the subject-specific data into 39 principal components. Next, reduced data of all subjects were concatenated across time and decomposed into 26 independent components using the infomax algorithm. To ensure estimation stability, the infomax algorithm was repeated several times in ICASSO (http://research.ics.tkk.fi/ica/icasso/), and the most central run was selected and analyzed further. Finally, participant-specific spatial maps and time courses were obtained using the GICA back reconstruction approach.

**Figure 1:**
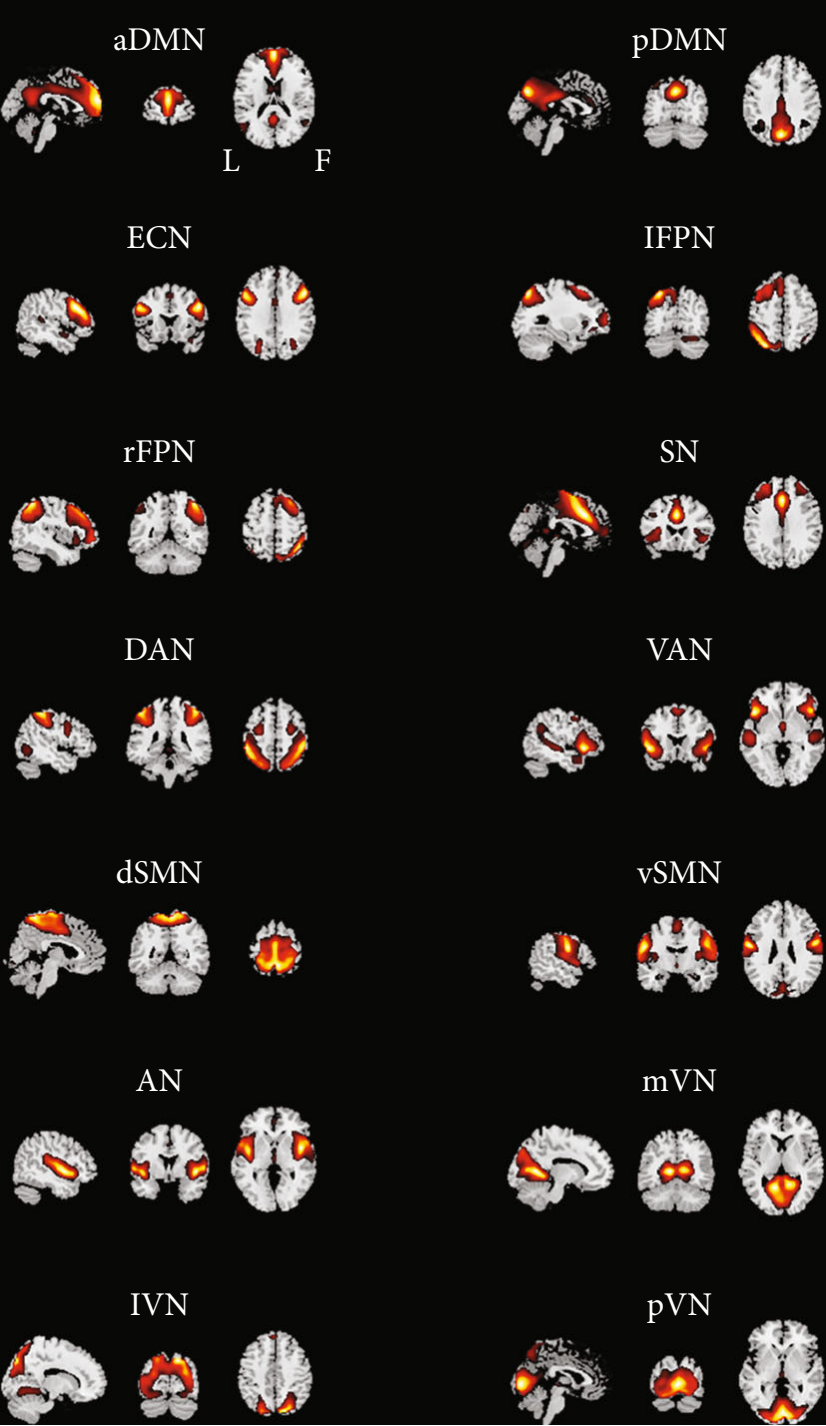
Spatial maps of 14 selected independent components. Abbreviations: aDMN: anterior default mode network; pDMN: posterior default mode network; ECN: executive control network; lFPN: left frontoparietal network; rFPN: right frontoparietal network; SN: salience network; DAN: dorsal attention network; VAN: ventral attention network; dSMN: dorsal sensorimotor network; vSMN: ventral sensorimotor network; AN: auditory network; mVN: medial visual network; lVN: lateral visual network; pVN: posterior visual network; L: left; R: right.

We identified as functional networks several independent components that had peak activations in gray matter; showed low spatial overlap with known vascular, ventricular, motion, and susceptibility artifacts; and exhibited primarily low-frequency power. This selection procedure resulted in 14 functional networks out of the 26 independent components obtained (Figure 1): anterior and posterior default mode networks (aDMN and pDMN); executive control network (ECN); left and right frontoparietal networks (lFPN and rFPN); salience network (SN); dorsal and ventral attention networks (DAN and VAN); dorsal and ventral sensorimotor networks (dSMN and vSMN); auditory network (AN); and medial, lateral, and posterior visual networks (mVN, lVN, and pVN).

### 2.7. Internetwork Functional Connectivity Analysis

Before internetwork functional connectivity calculation, the following additional postprocessing steps were performed on the time courses of selected functional networks: (1) detrending linear, quadratic, and cubic trends; (2) despiking detected outliers; and (3) low-pass filtering with a cut-off frequency of 0.15 Hz. Then, internetwork functional connectivity was estimated as the Pearson correlation coefficients between pairs of time courses of the functional networks, resulting in a symmetric 14 × 14 correlation matrix for each subject. Finally, correlations were transformed to *z*-scores using Fisher's transformation to improve the normality [32].

Our analysis fitted a linear regression model with internetwork functional connectivity as the dependent variables and serum lipid levels as the predictors, controlling for age, sex, and FD as nuisance covariates. For correction of the multiple comparisons, the results were corrected by false discovery rate (FDR) with a corrected significance level of *P* < 0.05. For internetwork functional connectivity showing correlations with serum lipid levels, we further examined their associations with working memory performance (3-back accuracy and reaction time) using partial correlations adjusting for age, sex, and FD.

To test whether the association between variables was mediated by other variables, mediation analysis was performed using the PROCESS macro (http://www.processmacro.org/) developed by Hayes [33]. The PROCESS uses an ordinary least squares path analytic framework to estimate direct and indirect mediation effects. In the mediation analysis model (Figure 4(a)), all paths were reported as unstandardized ordinary least squares regression coefficients, namely, total effect of *X* on *Y* (*c*) = indirect effect of *X* on *Y* through *M* (*a* × *b*) + direct effect of *X* on *Y* (*c*′). The significance analysis was based on 5000 bootstrap realizations, and the significance of indirect effects was assessed by bootstrap 95% confidence interval (CI). In the PROCESS analysis, a significant indirect effect is indicated when the bootstrap 95% CI does not include zero. In this study, only variables that showed a significant correlation with others were considered independent (serum limpid levels), dependent (working memory performance), or mediating (internetwork functional connectivity) variables in the mediation analysis. Age, sex, and FD were considered nuisance variables.

### 2.8. Intranetwork Functional Connectivity Analysis

Intranetwork connectivity was examined via the spatial maps, indexing the contribution of the time course to each voxel comprising a given component. Specifically, all participants' spatial maps for each functional network were entered into a random-effect one-sample *t*-test. Brain regions were considered to be within each network if they met a height threshold of *P* < 0.05 corrected for multiple comparisons using a family-wise error (FWE) and an extent threshold of 100 voxels. Next, we examined the correlations between intranetwork functional connectivity and serum lipid levels in a voxel-wise manner within each network using a multiple regression model while controlling for age, sex, and FD. Multiple comparisons were corrected using the cluster-level FWE method, resulting in a cluster defining threshold of *P* = 0.001 and a corrected cluster significance of *P* < 0.05, following current standard [34]. For intranetwork functional connectivity showing correlations with serum lipid levels, we employed the same procedures (i.e., partial correlation and mediation model) to conduct the subsequent analyses.

## 3. Results

**Figure 2:**
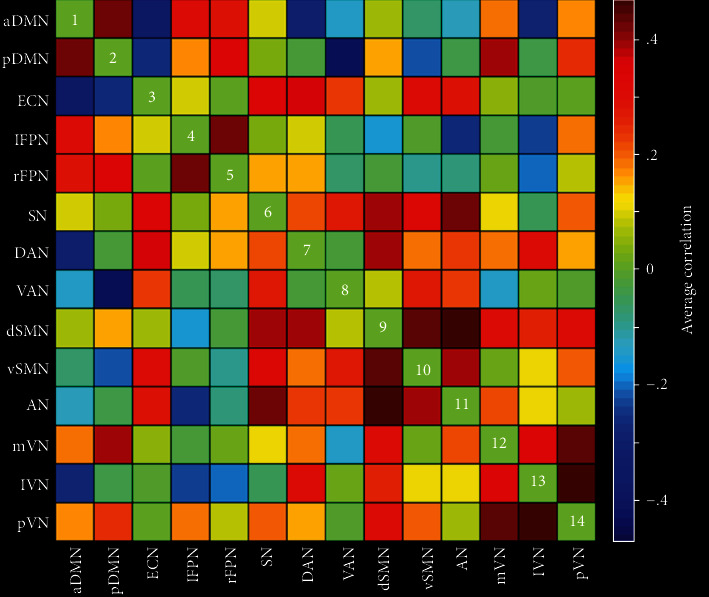
Internetwork functional connectivity matrix. Pairwise correlations between functional networks were averaged across subjects. Hot colors represent positive functional connectivity, and cool colors represent negative functional connectivity. Abbreviations: aDMN: anterior default mode network; pDMN: posterior default mode network; ECN: executive control network; lFPN: left frontoparietal network; rFPN: right frontoparietal network; SN: salience network; DAN: dorsal attention network; VAN: ventral attention network; dSMN: dorsal sensorimotor network; vSMN: ventral sensorimotor network; AN: auditory network; mVN: medial visual network; lVN: lateral visual network; pVN: posterior visual network.

**Figure 3:**
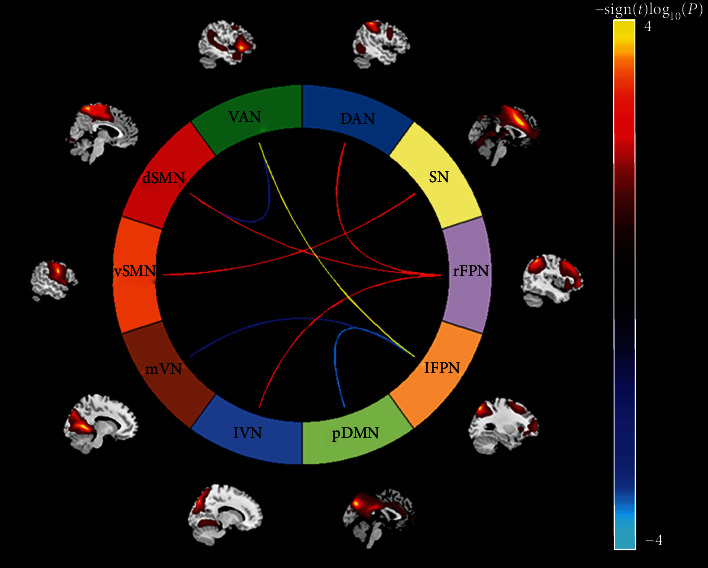
Associations between serum TG level and internetwork functional connectivity. Hot colors represent positive correlations, and cool colors represent negative correlations. Abbreviations: TG: triglyceride; pDMN: posterior default mode network; lFPN: left frontoparietal network; rFPN: right frontoparietal network; SN: salience network; DAN: dorsal attention network; VAN: ventral attention network; dSMN: dorsal sensorimotor network; vSMN: ventral sensorimotor network; mVN: medial visual network; lVN: lateral visual network.

Pairwise correlation patterns between functional networks are illustrated in Figure 2. Both positive and negative internetwork functional connectivity were observed. Linear regression analyses revealed significant correlations between serum TG level and internetwork functional connectivity (*P* < 0.05, FDR corrected) (Figure 3). Specifically, serum TG level was positively correlated with functional connectivity between lFPN and VAN (*t* = 4.00, *P* = 0.0001), between rFPN and DAN (*t* = 3.15, *P* = 0.0020), between rFPN and dSMN (*t* = 3.18, *P* = 0.0018), between rFPN and lVN (*t* = 2.96, *P* = 0.0036), and between SN and vSMN (*t* = 3.08, *P* = 0.0025), as well as negatively correlated with connectivity between pDMN and lFPN (*t* = −3.44, *P* = 0.0007), between lFPN and mVN (*t* = −2.92, *P* = 0.0040), and between VAN and dSMN (*t* = −3.04, *P* = 0.0028). However, there were no significant correlations between internetwork functional connectivity and serum levels of other lipid parameters (i.e., TC, HDL-C, and LDL-C).

**Figure 4:**
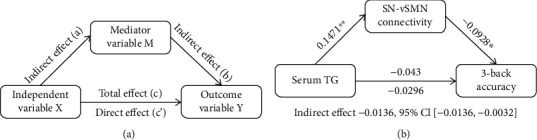
Conceptual diagram of mediation analysis. (a) Graphical representation of a mediation analysis model with one mediator. Total effect of *X* on *Y* (*c*) = indirect effect of *X* on *Y* through M (*a* × *b*) + direct effect of *X* on *Y* (*c*′). (b) The mediation analysis between serum TG level (*X*) and 3-back accuracy (*Y*), with SN-vSMN connectivity as the mediator (M). Path coefficients with *P* values (^∗^*P* < 0.05 and ^∗∗^*P* < 0.01, respectively). Abbreviations: TG: triglyceride; SN: salience network; vSMN: ventral sensorimotor network.

Partial correlation analyses revealed a significant negative correlation between SN and vSMN connectivity and 3-back accuracy (*pr* = −0.210, *P* = 0.009). This correlation was still significant when additionally controlling for educational level (*pr* = −0.203, *P* = 0.012). However, no significant correlation between internetwork connectivity and 3-back reaction time was present. Further, the mediation analysis model and the investigated variables are described in Figure 4. The relationship between serum TG level and 3-back accuracy was significantly mediated by SN-vSMN connectivity (indirect effect = −0.0136, SE = 0.0070, 95% CI: -0.0316, -0.0032).

Voxel-wise analyses of the spatial maps demonstrated no significant correlations between serum lipid levels and intranetwork connectivity within any of the functional networks.

## 4. Discussion

This is the first study to comprehensively investigate the associations between serum lipid profile and functional connectivity between and within large-scale networks in young adulthood. Our analyses revealed that higher serum TG level was associated with stronger internetwork functional connectivity of lFPN-VAN, rFPN-DAN, rFPN-dSMN, rFPN-lVN, and SN-vSMN and lower connectivity of pDMN-lFPN, lFPN-mVN, and VAN-dSMN. Among the TG-linked functional connectivity, greater SN-vSMN connectivity was correlated with poorer working memory performance. More importantly, mediation analysis further demonstrated that SN-vSMN connectivity was a significant mediator of the association between serum TG level and working memory performance.

By means of multimodal neuroimaging techniques, researchers have explored the relationships between serum lipid variables and multiple brain imaging parameters. For examples, using task-based fMRI, Gonzales and colleagues found that higher serum TC/HDL-C ratio related to reduced working memory-related activation intensity in the parietal and frontal regions in midlife [15]. In a resting-state fMRI study of nondemented elderly, higher serum TC was shown to associate with lower functional connectivity in the salience network yet stronger connectivity in the default mode network [16]. Another resting-state fMRI based on graph theory suggested that higher serum LDL-C level was associated with intensified age-related neural network decoupling (i.e., decreased network connectivity with age) [17]. By employing structural MRI, Ward et al. observed an association between lower serum HDL-C and reduced gray matter volume in the temporal, occipital, and parahippocampal regions in a sample of middle-aged and elderly people [12]. In addition, there is empirical evidence that serum lipids appear to be implicated in the neuropathological processes of some clinical conditions. For instance, a resting-state fMRI study of type 2 diabetes mellitus revealed that compared with target cholesterol patients, those with poorly controlled cholesterol (i.e., higher serum TC, LDL-C, and LDL-C/HDL-C ratio) exhibited aberrant hippocampus-middle frontal gyrus functional connectivity [4]. Gonzalez-Escamilla et al. reported that serum HDL-C level was positively correlated with the strength of neural-phase coupling and increased TG accompanied gray matter loss in the precuneus in mild cognitive impairment [13]. A recent structural MRI study demonstrated that gray matter reduction in the parietal, occipital, and cerebellar areas associated with increased serum TG and LDL-C in subjects with obesity [14]. Using surface-based morphometry analysis, Kinno et al. identified positive correlations between serum HDL-C level and gyrification indices of the insular and frontal opercular cortices in an elderly memory-clinic population [5]. Collectively, these prior reports point towards a negative effect of serum TC, TG, and LDL-C yet a protective effect of HDL-C on brain function and structure. By contrast, the current observation of positive and negative associations of serum TG level with connectivity between a set of large-scale functional networks highlights the prominent role of TG in modulating internetwork communication and coordination in young adulthood. This has led to some speculation that increased TG reflects an excessive accumulation of free fatty acid, which can damage neuron by inflammatory process [35]. Neuronal injury may in turn result in widely distributed dysfunctional connectivity along with compensatory neuroadaptive connectivity changes.

There is diverse and convergent evidence in support of a negative effect of serum TC, TG, and LDL-C yet a protective effect of HDL-C on cognitive function. For example, Stough and colleagues found that increases in TC and LDL-C were associated with decreased cognitive performance in recognition memory, working memory, and inhibitory processing in healthy elderly adults [1]. In a cross-sectional study of aging women, higher HDL-C was found to be correlated with better verbal learning and memory performance [6]. Parthasarathy et al. identified a negative effect of serum TG on executive function in nondemented aging adults, which was independent of vascular risks and cerebrovascular injury [2]. Even in a nonelderly sample, serum TC was found to be negatively associated with executive control and sustained attention [3]. For some clinical conditions, this was also the case. A recent study revealed that higher HDL-C was associated with better executive function in patients with diabetes mellitus [7]. Another study of type 2 diabetes mellitus reported that patients with poorly controlled cholesterol (i.e., higher serum TC, LDL-C, and LDL-C/HDL-C ratio) showed impaired attention and executive function relative to target cholesterol patients [4]. In an elderly memory-clinic population, memory function was found to be positively correlated with serum HDL-C level and negatively correlated with serum TG level [5]. These effects of serum lipid variables on cognition are also confirmed by some longitudinal studies. For instance, a cohort study in a large sample demonstrated that elevated serum TC, TG, and LDL-C in midlife was associated with greater 20-year cognitive decline [8]. In a 16-year longitudinal study, higher HDL-C and lower TG were identified to predict better maintenance of cognitive functions including verbal ability and perceptual speed in women rather than in men [9]. Another longitudinal study of an average follow-up of 21 years further uncovered a bidirectional relationship between serum TC and cognition, i.e., higher midlife TC was associated with poorer late-life episodic memory and category fluency, whereas more decreasing TC after midlife was related to poorer late-life episodic memory and psychomotor speed [10]. Our mediation analysis indicates that higher serum TG leads to stronger SN-vSMN functional connectivity, which in turn results in lower 3-back accuracy. This finding of a negative effect of serum TG on working memory via internetwork functional connectivity in healthy young adults not only may complement and extend previous literature on serum lipids-cognition relationships but also may help shed light on the neural mechanism underlying such associations.

There are several limitations that should be mentioned. First, our cross-sectional design does not allow inference on causality. Longitudinal studies with intervention targeted toward altering serum lipid levels are needed to establish the direction of causality. Second, since this study population was self-selected from a group of educated volunteers, the present findings may not be representative of the general population. Third, in view of the selection biases in identifying meaningful functional networks from ICA-derived components, there may be additional functional networks that we have not considered. Fourth, serum lipids may be affected by diet, especially a high-fat diet. However, we did not collect information about the recent diet of the participants, which prevents us from further investigating the effect of diet on our findings. Fifth, the cognitive function was measured by a 3-back task, which only assesses working memory. Future studies utilizing a more comprehensive measure involving multiple cognitive domains are much needed. Sixth, artifacts from cardiac and respiratory noise are prevalent in resting-state fMRI analyses. Thus, an advisable preprocessing step is to remove physiological noise from the data using simultaneously collected pulse and respiration data. However, physiological data were not collected in this study. Finally, we did not find any significant correlations between functional connectivity and serum levels of other lipid variables (i.e., TC, HDL-C, and LDL-C), and the reason for the null findings needs to be further explored. One possibility is that there may be a complex relationship between these lipid variables and functional connectivity beyond a simple linear correlation.

## 5. Conclusions

The present work provides novel preliminary evidence that serum TG can modulate large-scale internetwork functional connectivity in young adulthood, which may act as a mediator of the association between TG and working memory performance. These findings not only may expand existing knowledge regarding serum lipids-brain-cognition relationships but also may ultimately inform a translational conceptualization of how to improve cognitive function through regulating serum lipids.

## Figures and Tables

**Table 1:** Demographic, behavioral, and serum lipid data of 157 healthy participants.

Characteristics	Mean ± SD	Range
Gender (female/male)	77/80	—
Age (years)	22.3 ± 2.4	18-28
Education (years)	15.8 ± 1.9	12-20
Serum lipid parameters		
TC (mmol/L)	4.08 ± 0.75	2.57-7.06
TG (mmol/L)	0.99 ± 0.53	0.25-3.40
HDL-C (mmol/L)	1.51 ± 0.34	0.60-2.39
LDL-C (mmol/L)	2.21 ± 0.67	0.98-4.51
3-back task performance		
Accuracy (%)	72.1 ± 15.7	15-98.3
Reaction time (ms)	769 ± 175	230-1180
FD (mm)	0.12 ± 0.05	0.04-0.40

Abbreviations: SD: standard deviation; TC: total cholesterol; TG: triglyceride; HDL-C: high-density lipoprotein cholesterol; LDL-C: low-density lipoprotein cholesterol; FD: frame-wise displacement.

## Data Availability

The datasets generated for this study are available on request to the corresponding author.
